# miRNA-Mediated Relationships between Cis-SNP Genotypes and Transcript Intensities in Lymphocyte Cell Lines

**DOI:** 10.1371/journal.pone.0031429

**Published:** 2012-02-14

**Authors:** Wensheng Zhang, Andrea Edwards, Dongxiao Zhu, Erik K. Flemington, Prescott Deininger, Kun Zhang

**Affiliations:** 1 Department of Computer Science, Xavier University of Louisiana, New Orleans, Louisiana, United States of America; 2 Department of Computer Science, Wayne State University, Detroit, Michigan, United States of America; 3 Department of Pathology, Tulane University Health Sciences Center and Tulane Cancer Center, New Orleans, Louisiana, United States of America; 4 Tulane Cancer Center, Tulane School of Public Health and Tropical Medicine, New Orleans, Louisiana, United States of America; Institut Pasteur, France

## Abstract

In metazoans, miRNAs regulate gene expression primarily through binding to target sites in the 3′ UTRs (untranslated regions) of messenger RNAs (mRNAs). Cis-acting variants within, or close to, a gene are crucial in explaining the variability of gene expression measures. Single nucleotide polymorphisms (SNPs) in the 3′ UTRs of genes can affect the base-pairing between miRNAs and mRNAs, and hence disrupt existing target sites (in the reference sequence) or create novel target sites, suggesting a possible mechanism for cis regulation of gene expression. Moreover, because the alleles of different SNPs within a DNA sequence of limited length tend to be in strong linkage disequilibrium (LD), we hypothesize the variants of miRNA target sites caused by SNPs potentially function as bridges linking the documented cis-SNP markers to the expression of the associated genes. A large-scale analysis was herein performed to test this hypothesis. By systematically integrating multiple latest information sources, we found 21 significant gene-level SNP-involved miRNA-mediated post-transcriptional regulation modules (SNP-MPRMs) in the form of SNP-miRNA-mRNA triplets in lymphocyte cell lines for the CEU and YRI populations. Among the cognate genes, six including ALG8, DGKE, GNA12, KLF11, LRPAP1, and MMAB are related to multiple genetic diseases such as depressive disorder and Type-II diabetes. Furthermore, we found that ∼35% of the documented transcript intensity-related cis-SNPs (∼950) in a recent publication are identical to, or in significant linkage disequilibrium (LD) (p<0.01) with, one or multiple SNPs located in miRNA target sites. Based on these associations (or identities), 69 significant exon-level SNP-MPRMs and 12 disease genes were further determined for two populations. These results provide concrete *in silico* evidence for the proposed hypothesis. The discovered modules warrant additional follow-up in independent laboratory studies.

## Introduction

MicroRNAs (miRNAs) are short (∼*22 nt*), non-coding RNAs derived from genome-encoded stem loop precursors. As the post-transcriptional regulators of gene expression in metazoans, miRNAs primarily bind to the 3′ UTR sequences of messenger RNAs (mRNAs), usually resulting in translational repression or mRNA degradation [1], [2]. A highly confident canonical target site on a mRNA for miRNA binding typically holds a perfect Watson-Crick complementarity to the critical “seed region” (nucleotides 2–7 at the 5′-end) of miRNA(s), and may include an extension of one base at position 1 or 8 where either an adenine is paired to nucleotide 1 (7mer-A1 site) or a matching base is present for nucleotide 8 (7 mer-m8 site) of the miRNA seed. An 8-mer site can also be formed if the target site flanked by both the adenine at position 1 and the match at position 8 [3]. It is estimated that ∼30% of human protein-coding genes are regulated by miRNAs, where each miRNA can target approximately 200 transcripts and more than one miRNA can converge onto a single mRNA target [2], [4]. Numerous studies have shown that miRNAs can play fundamentally crucial roles in various biological functions, including animal and plant development as well as progression of human diseases [1], [2], [5], [6].

Single-nucleotide polymorphisms (SNPs) are the most abundant form (∼90%) of variation in the human genome. Basically, they occur when a single nucleotide in the genome (or other shared sequence) differs between members of a species or paired chromosomes in an individual. The recently released “NCBI dbSNP Build 135” database contains about 54 million SNPs from the human genome [7]. As the molecular markers of complex traits, SNPs have been widely investigated in humans, animals and plants [8], [9], [10], [11], [12], [13], [14], [15], [16]. Recent studies showed that cis-acting SNPs (cis-SNP markers), within or close to a gene itself, are predominant compared to copy number variations (CNV) in explaining the genetic variations of gene expression measures; and many of them are related to human diseases [17], [18], [19]. These associations are important in the prediction of individual predisposition to diseases in humans and the genetic evaluation of economic traits in crops and domestic animal species. Nevertheless, while the SNPs in protein-coding regions may directly determine the genetic variance of phenotype measures, most SNP markers identified in other regions are likely coincident with the actually recognized or unknown causal functional variants [20], [21], [22]. This implies that the mechanisms behind the genetic variations of many complex traits are still not clear, and remain an elusive challenge in genetics and related fields.

Compared to ORFs, the 3′ UTRs of human genes have higher SNP density [23]. According to the dbSNP database (Build 135) [7], over 334000 3′-UTR SNPs have been validated by at least one method or are included in the submission of the 1000 Genome Project [24]. This suggests a promising way to open these black boxes. This perception can be scrutinized from two aspects. First, it is well known that miRNAs regulate gene expression by binding to cis-regulatory regions of 3′-UTRs of genes. A nucleotide variation in 3′UTR sequences can alter the complementarity between a miRNA and the matched region in its target mRNA(s), thereby influencing the accessibility for miRNA binding. In this regard, those 3′UTR-SNPs may function as the causal elements for the variance of the expression of the target genes [25]. Second, because the alleles of different SNPs within a DNA sequence of limited length tend to be in strong linkage disequilibrium (LD) [26], the variants of miRNA target sites potentially serve as bridges linking the cis-SNP markers documented in the literature to the expression of the associated genes.

To date, several attempts have been made to explore the biological implications of miRNA target site polymorphism caused by SNPs [27]. Most efforts, using either *in vitro* or small-scale *in silico* methods, primarily focused on studying the targeted association between a specific genetic variant in miRNA target site(s) and a particular human disease. However, understanding the general regulatory mechanism of miRNAs in the overall gene regulation, especially when SNPs residing on miRNA-binding sites adds another layer of complexity, is also essential to the discovery of SNPs and miRNAs' interlacing functions in complex trait formations and gene regulation system.

In order to understand the regulatory mechanisms between SNPs, miRNAs and their target genes, we need to identify the functional modules and important patterns hidden in this complicated interactions. Two earlier studies, though not directly related to our work, are worth mentioning here. Bao et al. established a database of polymorphism (SNPs) in putative microRNA Target Site (PolymiRTS) and proposed a simple conceptual model to tie PolymiRTS to complex traits via cis-acting eQTL (the genetic loci regulating gene expression traits) [28]. The main limitation of their work is that miRNA gene expression profiles were not taken into account due to the lack of large-scale miRNA expression data at that time. Another study, conducted by Saunders et al., incorporated miRNA and mRNA expression data to identify novel biologically (especially evolutionarily) relevant miRNA target sites [29]. This work, however, relies on the co-expression of miRNA(s) and gene(s) in at least one of the five distinct tissues. Therefore, their findings cannot truly reflect the fundamental miRNA and mRNA interaction which can only be revealed through miRNA and mRNA expression quantities obtained from a specific biological process in the same or comparable cells or tissues.

**Figure 1:**
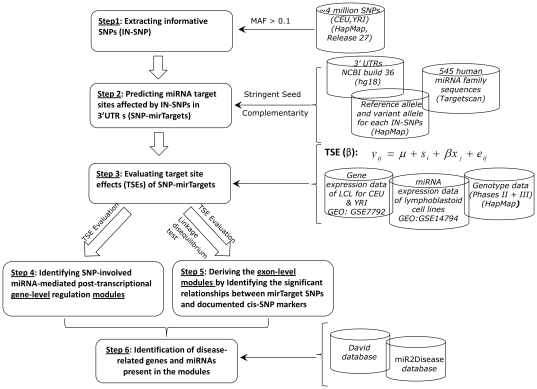
The schematic presentation of the study flow.

In this paper, following our preceding scrutinization, we provided the first piece of *in silico* evidence for the potential novel regulatory role of miRNA-target-site SNPs in associating the documented cis-SNP markers with the expression of miRNA target gene(s). By integrating multiple latest information sources, including SNP genotype data, human miRNA family information, gene expression and miRNA expression profiles on similar cell lines, we first identified 21 significant gene-level SNP-involved, miRNA-mediated post-transcriptional regulation modules (SNP-MPRMs) in the CEU (US residents of Northern and Western European descent) and YRI (Ibadan, Nigeria) populations. A linear model was proposed to estimate the statistically significant miRNA target site effect (TSE) on the target gene(s). Moreover, by calculating the pair-wise LDs, we related the SNPs located in the miRNA target sites to the documented cis-acting SNPs for the same LCL (lymphocyte cell line) samples [13], resulting in 69 significant exon-level SNP-MPRMs. Evaluating the discovered modules by using the literature and functional annotation tool DAVID suggests that some genes in the modules are involved in several types of human diseases. These modules are worthy of further laboratory testing due to their biomedical implications. **Figure 1** summarizes the scheme of our study flow, and the details of each step are described in the Results and Method sections.

## Results

### IN-SNPs involved miRNA target site prediction and site verification

The total number of documented single nucleotide polymorphisms in the human genome now totals 54 million. In this study, we focused on the ∼4-million SNPs present in the CEU or YRI population genotyped in the international HapMap project. This restricted SNP set was further refined according to the minor allele frequencies (MAF), and only those SNPs with MAFs over 0.1, called informative SNPs (IN-SNPs) hereafter, were kept for further analysis. Such selection is based on our preliminary evaluation of the available resources for detecting biologically significant SNPs located in miRNA target sites. More specifically, the data preprocessing was determined by two factors. First, the microarray datasets [8] analyzed in this study are limited in sample size. Second, in such cases, if the MAF of a SNP is below 0.1, the statistical power and reliability will be low in the subsequent genetic association analysis [13].

**Figure 2:**
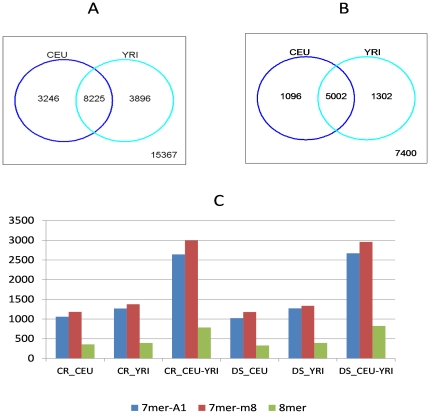
The summary of the predicted SNP-involved canonical miRNA target sites. **A:** Venn diagram comparing the distribution of the predicted SNP-involved miRNA target sites between CEU and YRI populations. **B:** Venn diagram comparing the distribution of genes, each of which contains at least one SNP-involved miRNA target sites, between CEU and YRI populations. **C:** The distribution of the predicted SNP-involved miRNA target sites with respect to population (CEU and YRI) and site mutation type (DS: disrupted sites and CR: created sites).

Using the procedure presented in the Material and Methods section, we identified the miRNA target sites affected by all IN-SNPs. These SNP-involved target sites were further categorized according to the three canonical site types (i.e. 7mer-A1, 7mer-m8 or 8mer) and the two site mutations - disrupted or created sites as defined in the Methods section. As shown in **Figure 2A**, among the ∼15400 IN-SNPs-involved miRNA target sites, more than half of them occur in both CEU and YRI sample sets. These target sites are located in the 3′UTR regions of the transcripts of 7400 genes, two thirds of which are shared by the two populations (**Figure 2B**). Functional enrichment analysis using the DAVID tool [30], [31] showed that four genetic disease classes, i.e. colorectal cancer (n = 106, FDR = 3.0%), blood pressure & arterial (n = 35, FDR = 6.3%), schizophrenia (n = 143, FDR = 9.6%), and atherosclerosis & coronary (n = 80, FDR = 22%), are over represented by these genes.

A closer examination of the site distribution with respect to population and site mutation type (**Figure 2C**) shows that 7mer-m8 target sites are a little more prevalent than 7mer-A1 sites. The 8mer target sites only account for 13–15% of the entire identified sites, much lower than the ratio (∼25%) calculated for the conserved target sites as reported by TargetScan [32]. According to [3], for the same miRNA-mRNA pair, 8mer sites tend to have higher mean efficiency in post-transcriptional regulation than 7mer target sites. Although the proportion of more biologically relevant 8mer sites in the non-conserved IN-SNP-involved target sites is much lower than that from the conserved target sites, these non-conserved SNP-present sites, once confirmed by biological experiments, will be more significant and informative than the validated conserved sites since they can potentially explain the genetic variance of some complex traits and even disease predispositions between and within populations.

**Figure 3:**
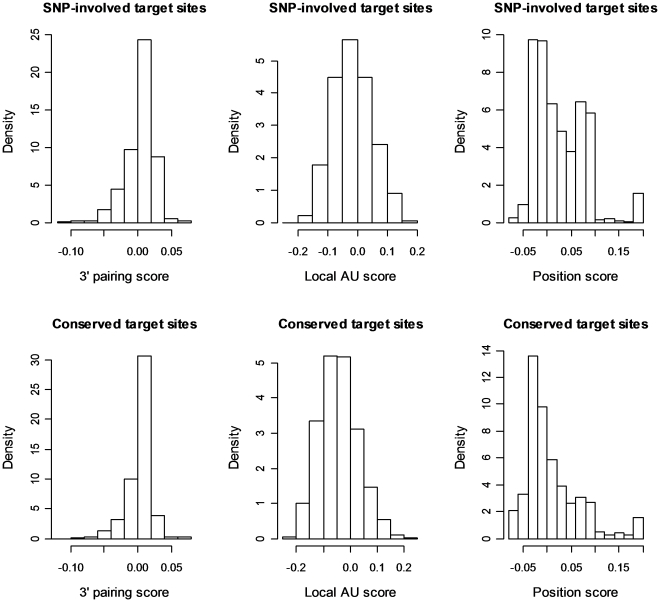
The density distribution comparisons of the context scores for the predicted SNP-involved miRNA target sites (non-conserved) and the conserved target sites reported by TargetScan. A target site with the score(s) deviating from zero to the negative direction is considered to be highly confident.

It has been recognized that the mechanism for a miRNA recognizing its target transcript(s) is far more complicated than the simple seed match rule, implying that some target sites preliminarily identified in this way can be spurious [2]. Therefore, nearly all of the published miRNA target prediction tools employed additional biological information beyond the miRNA and mRNA sequences to generate confidence scores and rank the candidate target sites accordingly [4], [6], [33], [34], [35], [36], [37]. Grimson et al. [3] proposed three biological-experiment-based context scores that have been integrated into TargetScan. Those scores are 3′ paring contribution, local AU contribution and position contribution. According to their definitions, a small negative value indicates high confidence. Using the released Perl program, we first calculated the three scores for ∼6000 predicted miRNA target sites created by substituting reference alleles with “other alleles” in CEU population, and then compared the scores with those calculated from the conserved target sites. **Figure 3** presents the density distributions of the two score sets. Statistical analysis using Wilcoxon rank-sum test demonstrated that the difference between these two site categories was not significant (p>0.05) in 3′ pairing contribution, but were extremely significant (p<2.2e-16) in local AU and position contributions. The SNP-involved target sites had higher means for the latter two contributions. This result is expected due to the following two reasons. First, we used the stringent seed match rule as the only criterion to identify the pool of potential miRNA target sites for the studied miRNA families. Second, different from those conserved target sites, the sites under investigation are no longer conserved because of the involvement of SNPs. Nevertheless, the substantial existence of IN-SNP-involved target sites with very low context scores also demonstrated that some predicted sites were of high confidence.

### Candidate IN-SNP-involved miRNA post-transcriptional regulation modules in LCLs

To gain more biological insight from the discovered SNP-miRNA target sites, we further characterized the potential SNP-involved miRNA-mediated post-transcriptional regulation modules (SNP-MPRMs). Such a module can reflect the modification of the transcript intensity of a target gene due to the SNP presence in the miRNA binding site, and could be formulated as a SNP-miRNA-mRNA triplet. Moreover, the module of this type can presumably reveal the SNP-involved, miRNA-mediated causative relationship between the documented cis-SNP markers and the differential expression of the target genes.

This work was achieved through the integration of genomic and transcriptomic information by defining and calculating the target site effects (TSEs) of expressed miRNA genes on the transcript intensities of their target genes. Specifically, TSE was estimated with a linear model as defined in the Material and Methods section. We obtained the gene expression data of 176 lymphocyte cell lines (LCL) for CEU and YRI populations from GEO (GSE7792) [8], and preprocessed them through the method described in [13]. The genotype data was downloaded from the database of International HapMap Projects (Phase II+Phase III) [38]. The evidence for the existence of miRNAs in lymphocyte cell were extracted from another GEO dataset (GSE14794) in which EBV transformed lymphoblastoid cell lines were measured from the peripheral blood lymphocytes of Caucasian men [39]. Details on the definition and calculation of TSE, the employed gene expression data, and the identification of expressed miRNAs are described in the Material and Methods section.

**Figure 4:**
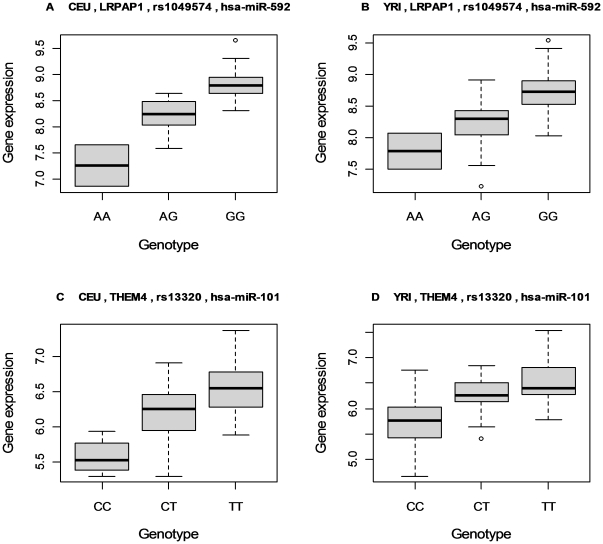
Box plots illustrating the additive pattern of miRNA target site effect (TSE) on gene expression. The upper plots ***A*** **and** ***B*** **:** Gene LRPAP1 has SNP rs1049574 in the 3′ UTR region. Based on the reference sequence hg18, the original nucleotide guanine (G) is not involved in any miRNA target site. The alternative nucleotide adenine (A) leads to the creation of a novel target site for miR-592. As a result, a sample of genotype GG, GA or AA has zero, one or two miRNA target sites due to this SNP, respectively. As shown in these two plots, the TSE in this created-target-site example well reflects an additive pattern for the post-transcriptional regulation of the target gene by the cognate miRNA in both populations. The lower plots ***C*** **and** ***D*** **:** gene THEM4 has SNP rs13320 in the 3′ UTR region. Based on the reference sequence hg18, the original nucleotide cytosine (C) is involved in a target site of miR-101. The alternative nucleotide thymine (T) leads to the disruption of the target site. As a result, a sample of genotype CC, CT or TT has two, one or zero miRNA target site due to this SNP, respectively. As shown in these two plots, the TSE in this disrupted-target-site example also well reflects an additive pattern for the post-transcriptional regulation of the target gene by the cognate miRNA in both populations.

Each SNP-involved miRNA target site corresponds to a unique SNP, one or multiple mRNA(s) and one miRNA family. Using the above genotype and transcript intensity information, we scanned all of the predicted SNP-involved miRNA target sites (∼15000) and calculated their corresponding TSEs. 17 and 9 candidate SNP-MPRMs were identified in CEU and YRI populations, respectively (**Tables 1** **,** **2**). The module determination was principally based on the widely-accepted miRNA post-transcriptional regulation theory [2]. First, for each module, the miRNA gene(s) should be expressed in lymphoblastoid cell lines and the target gene should be expressed in LCL samples. Second, the estimated TSE is negative with the adjusted p-value <0.05. Finally, the TSE should well reflect an additive pattern for the post-transcriptional regulation of a target gene by the cognate miRNA, which was illustrated in **Figure 4** on genes LRPAP1 and THEM4 but also largely hold on the other 18 genes (**Tables 1** **,** **2**).

**Table 1:** The SNP-involved, miRNA-mediated post-transcriptional regulation modules (SNP-MPRMs) in the CEU population.

Gene	Chr	Mutation	SNP	TSE	Adj.p	miRNA
BTN3A2	chr6	CR	rs2073531	−0.872	8.15E-15	326, 9
C17orf68	chr17	CR	rs3027246	−0.145	2.26E-03	542-3p
DGKE*	chr17	CR	rs1992554	−0.230	3.09E-02	545, 15a, 15b, 16, 195, 424, 497
GNA12*	**chr7**	**CR**	**rs11354**	**−0.223**	**1.64E-02**	**596**
KLF11*	chr2	CR	rs7632	−0.329	3.95E-02	512-5p
LRPAP1*	**chr4**	**CR**	**rs1049574**	**−0.599**	**1.81E-13**	**592**
MLF1IP	chr4	CR	rs3184982	−0.356	4.03E-02	18a, 18b
MMAB*	chr12	CR	rs877710	−0.137	4.39E-02	564
NDUFA10	chr2	CR	rs8369	−0.187	1.22E-02	15a, 15b, 16, 195, 424, 497
**NSUN4**	**chr1**	**CR**	**rs41534051**	**−0.153**	**2.77E-02**	**7**
**TOMM22**	**chr22**	**CR**	**rs1056610**	**−0.316**	**3.06E-05**	**451**
CTTNBP2NL	chr1	DS	rs3762332	−0.259	2.71E-02	148a, 148b, 152
CTTNBP2NL	chr1	DS	rs3795821	−0.248	4.39E-02	643
EMB	chr5	DS	rs2883164	−0.231	4.63E-02	21
ENTPD1	chr10	DS	rs2226163	−0.162	7.85E-03	617
RCBTB1	chr13	DS	rs1046028	−0.215	2.13E-02	643
**THEM4**	**chr1**	**DS**	**rs13320**	**−0.465**	**3.13E-08**	**101**

**SNP:** The SNP located in a predicted miRNA targets site in the 3′ UTR region of the corresponding gene; **Mutation:** DS indicates that the target site predicted from the reference sequence (**hg18**) is disrupted due to the nucleotide substitution, and CR indicates that a novel target site is created due to the substitution; **TSE:** miRNA target site effect on gene expression level; **adj.p**: FDR corrected p-value for TSE; **miRNA:** MiRBase ID of a human miRNA with the corresponding SNP in the predicted target site. The common prefix “hsa-mir(let)-” in the IDs is omitted. The rows in bold indicate the regulation modules shared in both CEU and YRI populations. See the method section for the calculation of TSE. Disease genes are indicated by asterisks (*).

**Table 2:** The SNP-involved, miRNA mediated, post-transcriptional regulation modules (SNP-MPRMs) in the YRI population.

Gene	Chr	Mutation	SNP	TSE	adj.p.	miRNA
CHI3L2	chr1	CR	rs1077059	−1.407	1.54E-05	591
GNA12*	**chr7**	**CR**	**rs11354**	**−0.203**	**1.18E-02**	**596**
LRPAP1*	**chr4**	**CR**	**rs1049574**	**−0.411**	**7.79E-04**	**592**
**NSUN4**	**chr1**	**CR**	**rs41534051**	**−0.175**	**2.90E-02**	**7**
**TOMM22**	**chr22**	**CR**	**rs1056610**	**−0.286**	**1.33E-05**	**451**
ZSWIM7	chr17	CR	rs11654	−0.182	8.92E-03	580
ALG8*	chr11	DS	rs616892	−0.148	4.40E-02	583
**THEM4**	**chr1**	**DS**	**rs13320**	**−0.437**	**1.97E-06**	**101**
VRK3	chr19	DS	rs16981592	−0.185	1.41E-02	365

**SNP:** The SNP located in a predicted miRNA targets site in the 3′ UTR region of the corresponding gene; **Mutation:** DS indicates that the target site predicted from the reference sequence (**hg18**) is disrupted due to the nucleotide substitution, and CR indicates that a novel target site is created due to the substitution; **TSE:** miRNA target site effect on gene expression level; **adj.p**: FDR corrected p-value for TSE; **miRNA:** the MiRBase ID of a human miRNA with the corresponding SNP in the predicted target site. The common prefix “hsa-mir(let)-” in the IDs is omitted. The rows in bold indicate the regulation modules shared by both CEU and YRI populations. See method section for the calculation of TSE. Disease genes are indicated by asterisks (*).

Among the discovered modules, five SNP-MPRMs were present in both populations with some differences in the TSE estimates. The target genes cognate to these modules include GNA12, LRPAP1, NSM4, TOMM22 and THEM4. Based on DAVID database [30], [31], of the total 20 genes in the two significant lists, six genes are related to human genetic diseases or biologically important quantitative traits. They are ALG8 related to congenital disorder of glycosylation, DGKE related to diversity of adult human height, GNA12 related to major depressive disorder, KLF11 related to type-II diabetes, MMAB related to dyslipidemia and coronary artery disease, and LRPAP1 related to Alzheimer's Disease and many other diseases. Besides the study of disease gene involvement in the discovered modules, we also studied the presence of disease-related miRNAs. According to [40], [41], 29 disease-related miRNAs were contained in the found modules, and 15 of them are associated with cancers (see **Table S1**).

It is worthy of note that some ambiguity exists in the discovered SNP-MPRMs. For instance, the module of gene DGKE, which plays an important role in signal transduction [42], is related to four human miRNAs within the same family, i.e. hsa-Mir- 15a, -15b, -16, -195, -424, and -497; Two SNP-MPRMs, one involving hsa-mir-643 and the other related to hsa-mir-148a, -148b and -152, were identified for gene CTTNBP2NL with almost equivalent TSEs but different SNPs. This ambiguity may generally be ascribed to two reasons. First, the same miRNA family shares a common seed sequence and our target prediction algorithm is primarily based on the seed match criterion. Second, more than one SNP-involved miRNA target sites could exist in the 3′UTR region of the same gene. At the expense of the potential false determination, such multifaceted modules could be further refined according to the 3′ pairing score or other related criteria, which are sometimes differentiated across individual miRNAs even in the same family.

### Variants of IN-SNP-involved target sites as bridges linking documented cis-SNP markers to transcript intensity

The associations between SNPs and the expression of the genes which those SNPs are located in or close to have been frequently reported over the past years [9], [13], [17]. However, the identified cis-acting SNPs, especially those obtained from the genome-wide scan, are not always the real causal regulators [20], [21]. In other words, the reported associations may be due to the coincidence of the molecular markers with other known or unknown genomic variants. In a study on the effects of single nucleotide substitutions on the alternative splicing of mRNA transcripts, Fraser and Xie [13] showed that over half of statistically significant SNPs are located within 30 kilo-bases from the affected transcript probe-sets (exons). Furthermore, they found that, among the top 20 exons (ranked by p-values), 14 are located in the 3′ UTR regions of the cognate genes. As they pointed out, these results suggested the participation of miRNA-binding related genomic variants in determining exon-level transcript intensity or mRNA splicing. However, the underlying mechanism still remains to be clarified. A further logical hypothesis would be that, for an association with such characteristics, the SNP marker may be either located in the binding site(s) of an expressed miRNA in the 3′ UTR of the target gene, or in strong linkage disequilibrium (LD) with one or multiple miRNA-target-site SNPs.

**Figure 5:**
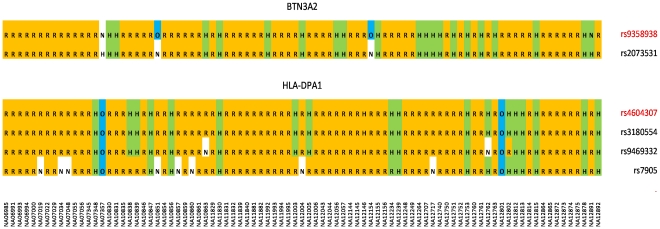
The illustration of the identity and association between SNPs in miRNA target sites and the documented SNP markers. In each panel (corresponding to a gene), the first row indicates the cis-associated SNP for the exon-level transcript level (a ratio) identified in [13], and the other rows each indicates a SNP related to a target site of an expressed miRNA in the EBV transformed lymphoblastoid cell lines. The letters in the shaded cells represented the recoded genotypes using the proposed method in this paper. R (highlighted in yellow) represents a (single-based) homozygous genotype with the two reference alleles as in hg18. H (highlighted in green) represents a heterozygous genotype with one reference allele and one alterative allele. O (highlighted in blue) represents a homozygous genotype with the two alternative alleles, and N (not shaded) represents an unknown genotype.

Using the proposed technique presented in the Material and Methods section, we recoded the genotypes of the miRNA-target-site SNPs of the addressed HapMap LCLs. The identity (or association) between the documented cis-SNP markers [13] and the miRNA-target-site SNPs identified in our study was illustrated in **Figure 5** **.** The LDs were first calculated as the squares of the Pearson correlation coefficients between SNPs, and then tested with the Chi square analysis. Among the 951 cis-acting SNPs, 41 (4.3%) were located in miRNA target sites of the cognate genes, and 287 (30.2%) were in significant linkage disequilibrium (p<0.01) with one or multiple target-site SNPs. Similar to the previous section, we first computed the target site effects of the expressed miRNAs on the transcript measures. Then, we selected the SNP-miRNA-exon triplets according to the signs and significance levels of the TSEs. With a ∼40% overlap, 57 and 56 significant (adj.p<0.05) exon-level SNP-MPRMs with negative TSEs were obtained for the CEU and YRI populations, respectively. In sum, 69 exons (of 67 genes) were involved in the two sets of SNP-MPRMs, where the reference alleles of each SNP pair are linked in coupling phase except for SNPs rs226201 and rs3738 (on gene RPS23) linked in repulsion phase. Ranked by the statistical significance, the top 20 modules for each population were summarized in **Tables 3** **and** **4**, and the entire module sets were presented in **Tables S2 and S3.** These modules not only provide solid *in silico* evidence for the hypothesis tested in this study, but also suggest that the miRNA-mediated post-transcriptional regulation mechanism may permit the partial degradation, starting from the target site, of a target mRNA sequence. In other words, miRNAs may only lead to the degradation of the individual exon (of a gene) close to their target site.

**Table 3:** The identity and association of top 20 miRNA-target-site SNPs with documented SNP markers in the CEU population.

Gene	Ps	SNP1	SNP2	LD 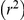	TSE	Adj.p	miRNA
BTN3A2	2899310	rs9358938	rs2073531	1.000	−1.427	5.93E-11	326, 9
C3orf19	2612007	rs9830735	rs11709673	1.000	−0.334	6.92E-07	657
CARKD	3501450	rs330563	rs11551105	0.427	−0.521	2.10E-10	138
CARKD	3501450	rs330563	rs330564	1.000	−0.393	8.80E-10	335
CCT2	3421668	rs4761246	rs7200	1.000	−0.098	1.71E-10	425
CHMP6	3737668	rs1128705	rs1128687	0.975	−0.246	7.27E-07	564
**CYTH1**	**3772526**	**rs6728**	**rs6728**	**1.000**	**−0.165**	**3.16E-08**	**10a**
DDTL	3939619	rs575959	rs1006771	0.840	−0.106	1.65E-08	221, 222
DOCK11	3988474	rs17326758	rs2379118	0.717	−0.760	8.80E-10	33b
**GTPBP5**	**3892544**	**rs2184161**	**rs2184161**	**1.000**	**−0.209**	**8.71E-08**	**324-5p**
HEATR1	2462521	rs2794766	rs6989	0.866	−0.091	2.18E-07	186
HLA-DPA1	2950342	rs4604307	rs3180554	1.000	−0.174	4.54E-25	522
HLA-DPA1	2950342	rs4604307	rs7905	0.708	−0.110	1.27E-07	191
KLHL5	2724198	rs3733279	rs1982009	1.000	−0.337	9.92E-08	500
KLHL5	2724198	rs3733279	rs3733275	1.000	−0.345	3.03E-07	570
NAA38	3020824	rs10085904	rs12537795	1.000	−0.163	2.01E-10	767-5p
NAA38	3020824	rs10085904	rs1061731	0.893	−0.144	7.43E-08	603
PDIK1L	2326294	rs17257113	rs17257120	1.000	−0.533	7.35E-13	591
PITRM1	3274176	rs2388556	rs12678	0.931	−0.131	5.83E-07	432, 768-5p
RPS23¶	2865071	rs226202	rs3738	0.988	−3.916	2.47E-09	190

**Ps:** the Affymatrix exon array transcript probe set; **SNP1:** the SNP marker documented in [13]; **SNP2:** the SNP located in a predicted miRNA targets site in the 3′ UTR region of the corresponding gene; **LD:** the linkage disequilibrium value 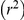 between **SNP1** and **SNP2**; **TSE:** the miRNA target site effect; **Adj.p:** the FDR corrected p-value for **TSE**; **miRNA:** the MiRBase ID(s) of a human miRNA with the **SNP2** in the predicted target site. The common prefix “hsa-mir-” in the IDs is omitted. In the rows with bold fonts, the **SNP2** is same as the **SNP1**. See method section for the calculations of **LD** and **TSE**. The modules are first ranked and selected by the significance level indicated by **adj.p**, and then presented by the alphabetic order of the gene names.

¶ The reference alleles of SNP1 and SNP2 are linked in repulsion phase.

**Table 4:** The identity and association of top 20 miRNA-target-site SNPs with documented SNP markers in the YRI population.

Gene	Ps	SNP1	SNP2	LD	TSE	Adj.p	miRNA
ARID5A	2494577	rs6576973	rs6576973	1.000	−0.186	9.69E-13	377
C3orf19	2612007	rs9830735	rs11709673	1.000	−0.194	9.55E-05	657
**C5orf15**	**2875930**	**rs10123**	**rs10123**	**1.000**	**−0.371**	**6.20E-10**	**642**
CCT5	2801555	rs2578645	rs699113	1.000	−0.102	6.20E-10	143
**CEACAM21**	**3834288**	**rs3745936**	**rs3745936**	**1.000**	**−0.580**	**2.68E-05**	**145**
**CHRAC1**	**3118459**	**rs10216653**	**rs10216653**	**1.000**	**−0.121**	**3.06E-06**	**328**
**CYTH1**	**3772526**	**rs6728**	**rs6728**	**1.000**	**−0.180**	**2.84E-05**	**10a**
**GTPBP5**	**3892544**	**rs2184161**	**rs2184161**	**1.000**	**−0.236**	**3.61E-09**	**324-5p**
HLA-DPA1	2950342	rs4604307	rs3180554	0.571	−0.205	8.88E-12	522
INO80C	3804023	rs3786394	rs11659769	1.000	−0.641	4.28E-12	570
KLHL5	2724198	rs3733279	rs1982009	0.918	−0.400	2.14E-05	500
KLHL5	2724198	rs3733279	rs3733275	1.000	−0.406	3.53E-05	570
**KYNU**	**2508576**	**rs1050950**	**rs1050950**	**1.000**	**−0.079**	**5.12E-05**	**432**
NAA38	3020824	rs10085904	rs12537795	1.000	−0.098	1.81E-06	767-5p
PDIK1L	2326294	rs17257113	rs17257120	1.000	−0.547	4.81E-07	591
PYGB	3880812	rs6083805	rs7020	1.000	−0.146	9.55E-05	196b, 624
TRUB1	3265505	rs11197010	rs7071789	1.000	−0.316	1.26E-08	495
VRK3	3868286	rs2304113	rs16981592	0.545	−0.095	1.13E-06	365
ZNF117	3004842	rs1524821	rs9638416	0.810	−7.303	1.41E-08	381
ZNF117	3004842	rs1524821	rs9638214	0.810	−6.807	7.09E-07	381

**Ps:** the Affymatrix exon array transcript probe set; **SNP1:** the SNP marker documented in [13]; **SNP2:** the SNP located in a predicted miRNA target site in the 3′ UTR region of the corresponding gene; **LD:** the linkage disequilibrium value 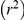 between **SNP1** and **SNP2**; **TSE:** the miRNA target site effect; **Adj.p:** the FDR corrected p-value for **TSE**; **miRNA:** the MiRBase ID(s) of a human miRNA with the **SNP2** in the predicted target site. The common prefix “hsa-mir-” in the IDs is omitted. In rows with bold fonts, the **SNP2** is same as the **SNP1**. See the method section for the calculations of **LD** and **TSE**. The modules are first ranked and selected by the significance level indicated by adj.p, and then presented by the alphabetic order of the gene names.

Using the DAVID tool [31], we identified 12 genes that are individually associated with one or multiple genetic disease(s) from these exon-level SNP-MPRMs. They are KYNU related to hypertension, HLA-DPA1 related to berylliosis and another 18 diseases, HLA-DQA2 related to rheumatoid arthritis, P2RY11 related to myocardial infarct, SYNGR1 related to schizophrenia and bipolar disorder, FIG. 4 related to amyotrophic lateral sclerosis 11 and Charcot-Marie-Tooth disease (type 4J), RTF1 related to congenital disorder of glycosylation (type In), CCT2 related to diversity of adult human height, CCT5 related to neuropathy and hereditary sensory; C5orf15 and PITRM1 related to conduct disorder and ADHD, and TMEM43 related to arrhythmogenic right ventricular dysplasia-5 (AVRD). Similarly, as summarized in **Table S1**, we identified 58 disease-related miRNAs were contained in the discovered exon-level modules, and 22 of them were associated with cancers.

## Discussion

In humans, miRNA target sites are mainly located in 3′ UTRs of the transcripts. Single nucleotide polymorphisms in 3′UTRs can alter the sequence complementarity between the miRNA(s) and mRNA(s), and hence eliminate existing target sites or generate novel target sites, suggesting an important mechanism for cis-regulation of gene expression. By integrating multiple recently published genomic and transcriptomic datasets for the samples of the HapMap projects, we evaluated the target site effects (TSEs) of expressed miRNAs on the transcript intensities of protein-coding genes in human lymphocyte cells. As a result, 21 gene-level and 69 exon-level SNP-MPRM modules were established for the CEU and YRI populations. To our knowledge, the collection of these modules forms the first bioinformatics knowledge basis for the further elucidation of the role of miRNA-target-site polymorphisms in target gene regulation. More specifically, the biological importance carried by these findings can be illustrated from the following aspects. (1) Most of the modules (>60%) contain created (CR) target sites which can't be identified if the polymorphisms are not taken into account; (2) More than a dozen of disease-related genes are involved in the discovered modules; (3) A substantial proportion (∼25%) of these modules are shared by the two populations, implicating a rather high level of confidence. For the time being, we don't find strong experimental evidence in the literature for these modules. This is no wonder because the study of miRNA polymorphisms is still in its infancy, and the published biologically confirmed SNP-involved miRNA-medicated relationships have yet been very scarce [43]. Therefore, the gene-level and exon-level modules discovered in this study can be treated as potential directional hypotheses that warrant further independent laboratory investigation. It is also worth noting that the absolute values of TSE for the discovered modules are relatively small, approximately less than one unit (fold change) in general. This implies that individual miRNA regulates the expression of the target genes in a tuning way. The observation also suggests that the modifications of miRNA target sites mainly contribute to the genetic variability of polygenic quantitative traits rather than individually result in dramatic physiological disorders.

After reviewing a few frequently cited miRNA-mediated associations primarily regarding polymorphisms and disorders [43], [44], we noted that this currently-completed work can be furthered consolidated by integrating more–omic information and other statistical measurements. First, while the analysis was based on the data of Human HapMap Project (phase II+III) in which the two populations each have ∼4 million SNPs, the recorded polymorphisms in human genome have accounted over 54 million. Furthermore, by considering the reliability of the statistical analysis, we studied only the SNPs with the MAFs over 0.1. As a result, it is very likely that some truly functional SNPs were left out due to the above two reasons. For example, SNP rs5186, which disrupted a target site of mir-155 on the transcript of gene AGTR [43], was not present in the analyzed LCL samples. The same situation was also observed in the case of SNP rs34764978, gene DHFR and mir-148a/-148b/-152 [45]. Second, we only considered the SNPs inside the canonical target sites recognized by the stringent seed match criterion without taking into account the SNPs within the atypical target sites or in other genomic regions. For example, on the 3′ UTR of cancer gene KIT, there is an atypical binding domain for miRNA-221. This target site sequence complements the nucleotides 2–5 and 7–8 of the miRNA. He et al. showed that SNP rs17084733 could disrupt this binding domain and resulted in a significant change of the transcript level [44]. Finally, as we did in this study, genome-wide association studies usually require the ordinary p-values to be adjusted in order to control the family wide error (FWE) or false discovery rate (FDR). This statistical correction will exclude some discovered modules with marginal significance from the reported list. For example, Wang et al. [46] demonstrated a direct association between miR-433 and SNP rs12720208 located in the 3′ UTR of gene FGF20 (a gene associated with an increased risk for Parkinson's disease). In our study, this SNP-miRNA-gene triplet was recognized but it wasn't selected into the module set since the corresponding TSE in CEU population was marginally significant (p = 0.055) as indicated by the ordinary p-value.

A serious challenge in analyzing the target site effects (TSEs), on which significant SNP-MPRMs were determined, is how to pre-process the microarray gene expression data. Because a SNP in a gene fragment can disrupt the hybridization of those probe(s) in the array and lead to false estimation of the expression level, we used the pre-processed data sets in [13] where probes containing double-documented SNPs were removed before the normalization and summarization. However, potential information loss due to such a preprocessing procedure is unavoidable. In fact, we noted its influence on the final results. For example, by analyzing the original data published at GEO, we could identify three SNP-MPRMs (adj. p<0.001) consisting of gene HLA-DPA1 (major histocompatibility complex, class II, DP alpha 1), a set of genotype consistent SNPs (rs9469332, rs3180554, rs7905) and three miRNAs (mir-448, -202, -522) in both CEU and YRI populations. However, when the same analysis was conducted on the preprocessed dataset, the three modules were no longer statistically significant (adj.p>0.05). Instead, the linkage disequilibrium analysis showed that the target sites containing these SNPs significantly (adj.p<1.0-e10) affected the transcript splicing of the gene (**Tables 3** **,** **4**). Therefore, there is a dilemma on using the pre-processed data and we will continue to investigate this problem.

Recently, RNA-seq data of HapMap LCL samples for the CEU and YRI populations have been generated and used to identify cis eQTLs [47], [48]. In particular, the authors in [48] found that many array-based eQTLs near the 3′ end of a gene are not present in the RNA-seq data. We herein repeated the SNP-MPRM scanning on these two digital gene expression datasets. As a result, 23 SNP-MPRMs were identified with an adjusted p-value <.05 (**Table S4**). Among them, 7 (∼30%) have counterparts in the array-based gene- or exon-level modules presented in the Result section. This indicates that our analysis is moderately robust to the technologies by which the gene expression levels are measured. However, we believe that the array datasets [8] analyzed in this paper are more appropriate for achieving our research objectives, i.e. to identify SNP-MPRMs and compare the two populations. Firstly, the array data have relatively larger sample sizes compared to the RNA-seq datasets (87 versus 60 for CEU, 89 versus 69 for YRI). This is extremely important in inferring the effects of SNPs with low minor allele frequencies. Second, the samples in the array data for the two populations were measured by the same authors on the identical technical platform following the same experimental protocols; therefore, the related batch effects could be greatly attenuated.

It is well known that the documented cis-SNP markers are not always the true causal factors to the variability of the associated phenotypes, including gene expression. In this regard, we hypothesized that some of them may function via linkage disequilibrium with SNPs in the miRNA target sites. Through further analyzing the results in [13], we found solid evidence for this hypothesis. This finding is important not only in comprehensively modeling the miRNA post-transcriptional regulation on the target genes, but also in deeply unraveling the genetic architecture and molecular mechanism of polygenic complex traits. In addition, different from the methods for selecting the most statistically informative SNPs [20], [21], [49], our result also suggests a novel strategy for the SNP association study. That is, when multiple SNP markers are synonymously related to the same trait, we can check if any of them is inside a miRNA target site in order to determine the true causal variant(s). For this purpose, we proposed a simple genotype recoding method to visualize the association between the SNPs of interest. A problem worthy of further note relates to the calculation of LD. For un-phased data, the required probability of haplotype AB of a pair of linked loci (suppose their bi-alleles are A/a and B/b, respectively) is usually estimated by one of the two iterative techniques, i.e. implementing an EM algorithm [50] or solving a cubic equation [49], [51], [52]. The first is theoretically attractive but cannot be guaranteed to converge to the global optimum [53]. In this study, we employed the second method but introduced a constraint on the interim estimate in the computation to avoid undesired results. An alternative approximation method we proposed here is that, without calculating the haplotype frequency, LD can be tested via evaluating genotype correlation. More precisely, for each locus, the three recoded genotypes R, H and O are first numbered as 1, 0 and −1, respectively. Then, Pearson correlation between the two loci is calculated as a pseudo LD measure and the significance is tested with Fisher' method or by an empirical distribution of the statistic established from the information of randomly sampled locus pairs. We tried this approximate method on the analyzed data and obtained similar results as those calculated using the algorithm described in the Material and Methods section. Due to its simplicity and no need for any prior parameters, the proposed method is worthy to be advocated in our opinion.

Great efforts have proven the influential role of *Cis*-acting *variants* in the expression of the host or adjacent genes. Various miRNA studies, on the other hand, have shown that accessibility of miRNA binding site is a crucial factor that controls the miRNA mediated regulation. SNPs that can contribute to alterations in the structure of miRNA binding site can thus influence the miRNA mediated regulation on target genes. In this study, by hypothesizing that miRNA-target-site polymorphism may represent a main mechanism for the individual and population variability of gene expression measures, and these site variants potentially link the documented cis-SNP markers to the expression of the associated genes, we performed a rigorous analysis to characterize genome-wide, SNP-involved, miRNA-mediated post-transcriptional regulation modules in lymphocyte cell lines. Our findings provided not only solid bioinformatic evidence for this hypothesis, but also two sets of statistically significant miRNA-SNP(s)-gene functional modules that will enable the targeted experimental studies.

## Materials and Methods

### Microarray gene expression data and its preprocessing

The raw data of gene expression profile of human lymphocyte cell lines (LCL) was deposited in the Gene Expression Omnibus repository (GEO, a MIAME compliant database) with the accession number GSE7792 [8]. This data, using Affymetrix Human Exon 1.0 ST arrays, measured 87 CEU and 89 YRI samples genotyped in International HapMap project. In the statistical analysis, we used a preprocessed dataset provided by Fraser and Xie [13]. The preprocess procedure was conducted as follows. First, the probes overlapping SNPs which had at least two-hit in the dbSNP database (release 126) [7], [54] were removed before analysis. Then, the probes in different arrays were normalized with the quantile method [55]. After that, the expression levels of probe sets and genes were summarized independently using the PLIER (probe logarithmic intensity error) algorithm implemented in the Affymetrix Power Tools (APT) software package. Finally, the normalized expression quantity of a probe set was determined by dividing the expression intensity with the intensity of the gene it belongs to. The detailed list of cis-SNP markers and the target probe-set (exons) within 100 kilo-bases were generated by editing the Supplemental Tables 3 and 4 in [13].

### Evidences for the existence of miRNAs in LCL samples

To our knowledge, no high-throughput miRNA expression profiling for the HapMap LCL samples has been published yet, though a recent publication reported that 58 LCL samples of CEU population had been measured with LNA microRNA Arrays [36]. In preparing this paper, we extracted the evidences for the existence (expression) of miRNAs in this type of cells from another GEO dataset (GSE14794) [39]. In this dataset, miRNA expression of 192 samples (including replicates) of 90 Epstein Barr virus (EBV)-transformed lymphoblastoid cell lines was measured with Illumina Human v1 MicroRNA expression beadchips. Since those lymphoblastoid cell lines are collected from the peripheral blood lymphocytes of Caucasian men, they are the closest substitute samples we can locate for the CEU population. To filter the miRNAs on the array, we employed a similar method as described in [39]. That is, based on the gene-level results generated from BeadStudio software, we first obtained the median detection p-values of the 736 miRNAs, and then corrected them with BH method [56]. After that, we selected 388 “expressed” miRNA genes with adj.p<0.05. Among them, 201 in the entire analyzed human miRNA set (N = 677) were kept for the subsequent study.

### Identification of SNP-involved miRNA target sites

Many computationally determined sets of miRNA target sites have been published on line [4], [32], [57]. In the employed prediction algorithm, the step of evaluating miRNA-target complementarity was generally included. To establish a large but relatively reliable pool of potential miRNA target sites, we implemented our lab-owned R program solely based on the criterion of stringent seed matching [2]. In particular, the canonical site motif on the 3′ UTR reverse complementary to the seed region (nucleotides 2–7(8)) of a miRNA was recognized by the *matchPattern* function contained in the Bioconductor *Biostrings* package [58]. We used three data sets at this step. They are (1) 545 human miRNA family sequences obtained from TargetScan website; (2) the allele frequency tables (Phase II+Phase III) downloaded from HapMap; and (3) 3′ UTR sequences of mRNAs retrieved from human genome assembly-18 (NCBI36). Using these data sets and the developed R codes, we predicted miRNA target sites containing the IN-SNPs through the following procedure. Two sequences, one centered on the reference allele and the other centered on the variant or other allele, were maintained for each IN-SNP. A site identified on the reference-allele sequence but not on the variant-allele sequence was marked as a disrupted (DS) target site, and a site predicted on the variant-allele sequence but not on the reference-allele sequence was marked as “created” (CR) target site. It should be noted that, only the SNPs with the MAF over 0.1 in CEU or YRI population were reserved in this study as IN-SNPs. Furthermore, the SNPs (accounting for ∼0.7% of the entire set of analyzed IN-SNPs) which followed the immediately preceding SNP with the distance less than 10 nt were also excluded from further analysis.

### Recoding SNP Genotypes

In the HapMap data (release 27), SNP genotypes are expressed in the bi-allelic form, such as A/C. In order to effectively estimate SNP-involved miRNA target site effect (TSE) on transcript intensity, and visualize the genotype correlation between two SNPs, we recoded the bi-allelic SNP genotype with a single capital letter. More specifically, “H” represents a heterozygous genotype with one reference allele and one alterative allele. “R” or “O” respectively denotes a homozygous (single-locus) genotype consisting of two reference or alternative alleles. According to this coding scheme, for instance, the genotypes of two loci or multi-loci can be expressed as RR, RRH, RHOR, and so on (see **Figure 5**). For convenience of writing, we call two SNPs “genotype consistent SNPs” if they have the recoded genotypes overlapped across all the individuals.

### Estimating SNP-involved miRNA target site effects (TSE)

The target site effect was estimated by a linear model as defined below.
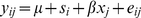
(1)where *y_ij_* is the log2 transformed transcript intensity (or ratio) of sample *j, μ* is the grand mean of all observations, *s_i_ (i = 1, 2)* is the sex effect, *β* is the TSE, *x_j_* is the number of “consistent allele(s)” (CA) of individual *j* at a specific target site, and *e_ij_* is the random noise. For a disrupted target site, R genotype has two CAs, H genotype has one CA and O genotype has zero CA. On the other hand, for a created target site, R, H and O genotypes have zero, one and two CA(s), respectively. The calculated p-value for a TSE was corrected with the BH method.

### Linkage disequilibrium (LD) calculation

LD was measured as the square of the correlation (r) between two SNP loci [59],
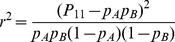
(2)where *p*_A_ and *p*_B_ are the probabilities of the reference alleles at the two loci, and can be directly estimated by their frequencies in the sample set; *P_11_* is the probability of the haplotype with two reference alleles. The statistic 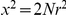 follows the Chi-square distribution with *df* = 1 under the null hypothesis of r^2^ = 0 [60].

The maximum likelihood (ML) estimation of *P_11_* was obtained through solving a cubic equation iteratively [51], [52]. That is,
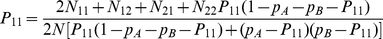
(3)In (3), *N* represents the total number of individuals. Based on the proposed genotype recoding scheme, *N_11_, N_12_, N_21_,* and *N_22_* represent the numbers of RR individuals, RH individuals, HR individuals and HH individuals, respectively. In the calculation, we also introduced a counting-based constraint defined as follows.
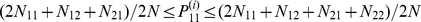
(4)where 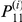 is the interim estimate in the *i*th iteration. The constraint is stricter than the limiting condition for valid roots described in [61], thus it can further alleviate the problem of convergence to a false optimum. Accordingly, the resulting LD (*r^2^*) can be guaranteed to be within the [0, 1] interval (see **Text S1**).

## Supporting Information

Table S1 The disease involvement annotation to the miRNAs in the predicted SNP-involved, miRNA-mediated post-transcriptional regulation modules listed in Tables 1–2 and Additional files 2–3.
(XLS)

Table S2 The identity and association of miRNA target site SNPs with documented cis SNP markers in the CEU population.
(XLS)

Table S3 The identity and association of miRNA target site SNPs with documented cis SNP markers in the YRI population.
(XLS)

Table S4 The RNA-seq data based prediction for SNP-involved, miRNA mediated, post-transcriptional regulation modules (SNP-MPRMs) in the CEU and YRI populations.
(DOC)

Text S1 The proof for the interval of the LD measure (r^2^) based on the haplotype probability estimated under the proposed constraint.
(DOC)
