# Supplementary material for: miRNA-Mediated Relationships between Cis-SNP Genotypes and Transcript Intensities in Lymphocyte Cell Lines
Source: PLoS One. 2012 Feb 14;7(2):e31429. doi: 10.1371/journal.pone.0031429 (PMC3279374; doi:10.1371/journal.pone.0031429)
Supplement: Table S4 — The RNA-seq data based prediction for SNP-involved, miRNA mediated, post-transcriptional regulation modules (SNP-MPRMs) in the CEU and YRI populations. (DOC) [file pone.0031429.s004.doc]

**Table S4**. The RNA-seq data based prediction for SNP-involved, miRNA mediated, post-transcriptional regulation modules (SNP-MPRMs) in the CEU and YRI populations a

| **Population** | **Gene** | **Chr** | **Mutation** | **SNP** | **TSE** | **adj.p** | **miRNA** |  |
| --- | --- | --- | --- | --- | --- | --- | --- | --- |
| CEU | NSUN4 | chr1 | CR | rs41534051 | -0.264 | 3.64E-02 | hsa-miR-7 | |
| CEU | MMAB | chr12 | CR | rs12817689 | -1.279 | 1.75E-02 | hsa-miR-519e | |
| CEU | STK10 | chr5 | CR | rs17074232 | -0.560 | 1.77E-04 | hsa-miR-200b, 200c, 429 | |
| CEU | HLA-DQB1 | chr6 | CR | rs9273448 | -0.577 | 6.81E-03 | hsa-miR-185 | |
| CEU | UQCRQ | chr5 | ds | rs17166297 | -0.420 | 4.56E-02 | hsa-miR-579 | |
|  |  |  |  |  |  |  |  |  |
| YRI | FAM20B | chr1 | CR | rs9725888 | -0.399 | 3.27E-02 | hsa-miR-542-3p, 431 | |
| YRI | EPRS | chr1 | CR | rs1061248 | -0.927 | 3.32E-02 | hsa-miR-581 | |
| YRI | DECR2 | chr16 | CR | rs1204504 | -0.685 | 3.26E-02 | hsa-miR-767-5p, 519 | |
| YRI | BSG | chr19 | CR | rs6757 | -0.654 | 4.56E-02 | hsa-miR-185 | |
| YRI | PYGB | chr20 | CR | rs7020 | -0.443 | 3.32E-02 | hsa-miR-196b, 624 | |
| YRI | GTPBP5 | chr20 | CR | rs2184161 | -0.491 | 5.17E-06 | hsa-miR-324-5p | |
| YRI | CEP63 | chr3 | CR | rs9809619 | -0.288 | 2.64E-02 | hsa-miR-539 | |
| YRI | MLF1IP | chr4 | CR | rs3184982 | -0.422 | 2.49E-02 | hsa-miR-18a | |
| YRI | PARM1 | chr4 | CR | rs9090 | -0.203 | 8.91E-03 | hsa-miR-132 | |
| YRI | ESYT2 | chr7 | CR | rs3763412 | -0.393 | 2.64E-02 | hsa-miR-190 | |
| YRI | ZNF117 | chr7 | CR | rs9638416 | -0.698 | 6.72E-05 | hsa-miR-381 | |
| YRI | THEM4 | chr1 | DS | rs13320 | -0.330 | 1.72E-02 | hsa-miR-101 | |
| YRI | SH2B3 | chr12 | DS | rs739496 | -0.105 | 1.93E-02 | hsa-miR-432 | |
| YRI | EARS2 | chr16 | DS | rs1468138 | -0.094 | 3.32E-02 | hsa-miR-494 | |
| YRI | C20orf194 | chr20 | DS | rs6037516 | -0.720 | 6.57E-03 | hsa-miR-766 | |
| YRI | GTPBP5 | chr20 | DS | rs2151511 | -0.349 | 6.72E-05 | hsa-miR-769-5p | |
| YRI | SUN2 | chr22 | DS | rs17032 | -0.213 | 4.26E-02 | hsa-miR-495 | |
| YRI | MLF1IP | chr4 | DS | rs14969 | -0.422 | 2.49E-02 | hsa-miR-505, 421 | |

^a^ Red marks the modules each of which has a counterpart among the array data based gene-level or exon-level modules. **SNP:** The SNP located in a predicted miRNA targets site in the 3’ UTR region of the corresponding gene; **Mutation:** DS indicates that the target site predicted from the reference sequence (**hg18)** is disrupted due to the nucleotide substitution, and CR indicates that a novel target site is created due to the substitution; **TSE:** miRNA target site effect on gene expression level; **adj.p**: FDR corrected p-value for TSE; **miRNA:** MiRBase ID of a human miRNA with the corresponding SNP in the predicted target site. YRI modules were discovered from the on-line normalized gene level expression dataset published by Pickrell et al (2010) . The CEU modules were discovered based on the short read data (SRA: ERP000101) distributed by Montgomery et al (2010) . We preprocessed the short read data through the following procedure. The short reads mapping against the human genome (hg18) and the computationally detection of exon-exon junctions were first performed by TopHat . Gene expression levels were then determined by Cufflinks with the UCSC GTF-format table of refSeq genes as the reference annotation. After that, we conducted the quantile normalization and log2 transformation. The genes with RPKM values equal to zero were filtered out before the normalization.
