# Supplementary material for: miRNA-Mediated Relationships between Cis-SNP Genotypes and Transcript Intensities in Lymphocyte Cell Lines
Source: PLoS One. 2012 Feb 14;7(2):e31429. doi: 10.1371/journal.pone.0031429 (PMC3279374; doi:10.1371/journal.pone.0031429)
Supplement: Text S1 — The proof for the interval of the LD measure (r2) based on the haplotype probability estimated under the proposed constraint. (DOC) [file pone.0031429.s005.doc]

**The proof for the interval of the LD measure (r^2^) based on the haplotype probability estimated under the proposed constraint**

According to the recoding scheme described in the **Material and Methods** section, there are 9 genotypes for any two SNPs A and B. Those genotypes are RR, RH, RO, HR, HH, HO, OR, OH and OO. Given a set of N individuals, the number of individuals for each genotype will be N_11_, N_12_, N_13_, N_21_, N_22_, N_23_, N_31_, N_32_ and N_33_, respectively. Then,

$p_{A}=\frac{1}{2N}(2N_{11}+2N_{12}+2N_{13}+N_{21}+N_{22}+N_{23})$ (5) $p_{B}=\frac{1}{2N}(2N_{11}+2N_{21}+2N_{31}+N_{12}+N_{22}+N_{32})$ (6)

Let

$U=(2N_{11}+N_{12}+N_{21}+N_{22})/2N$ , (7)

$L=(2N_{11}+N_{12}+N_{21})/2N$ (8)

When $D=P_{11}-p_{A}p_{B}>0$,

$\begin{matrix} r=\frac{P_{11}-p_{A}p_{B}}{[p_{A}p_{B}(1-p_{A})(1-p_{B})]^{\frac{1}{2}}} \\ \\ \text{ }\leq\frac{U-p_{A}p_{B}}{[p_{A}p_{B}(1-p_{A})(1-p_{B})]^{\frac{1}{2}}} \\ \\ \text{ }\leq\frac{min(p_{A},p_{B})-p_{A}p_{B}}{[p_{A}p_{B}(1-p_{A})(1-p_{B})]^{\frac{1}{2}}} \\ \\ \text{ }\leq1 \end{matrix}$ (9)

When $D=P_{11}-p_{A}p_{B}<0$,

$\begin{matrix} r=\frac{P_{11}-p_{A}p_{B}}{[p_{A}p_{B}(1-p_{A})(1-p_{B})]^{\frac{1}{2}}} \\ \\ \text{ }\geq\frac{L-p_{A}p_{B}}{[p_{A}p_{B}(1-p_{A})(1-p_{B})]^{\frac{1}{2}}} \\ \\ \text{ }\geq\frac{max(0,p_{A}+p_{B}-1)-p_{A}p_{B}}{[p_{A}p_{B}(1-p_{A})(1-p_{B})]^{\frac{1}{2}}} \\ \\ \text{ }\geq-1 \end{matrix}$ (10)
